# Newfound Hantavirus in Chinese Mole Shrew, Vietnam

**DOI:** 10.3201/eid1311.070492

**Published:** 2007-11

**Authors:** Jin-Won Song, Hae Ji Kang, Ki-Joon Song, Thang T. Truong, Shannon N. Bennett, Satoru Arai, Ninh U. Truong, Richard Yanagihara

**Affiliations:** * Korea University, Seoul, South Korea; † University of Hawaii at Manoa, Honolulu, Hawaii, USA; ‡ National Institute of Hygiene and Epidemiology, Hanoi, Vietnam

**Keywords:** Hantavirus, shrews, phylogeny, Vietnam, dispatch

## Abstract

Sequence analysis of the full-length medium segment and the partial small and large segments of a hantavirus, detected by reverse transcription–PCR in lung tissues of the Chinese mole shrew (*Anourosorex squamipes*) captured in Cao Bang Province, Vietnam, in December 2006, indicated that it is genetically distinct from rodentborne hantaviruses.

Insectivores (or soricomorphs) have been largely ignored as being important in the evolutionary dynamics of hantaviruses, despite the isolation of Thottapalayam virus (TPMV) from the Asian house shrew (*Suncus murinus*) (*1*,*2*) and the detection of hantavirus antigens in tissues of the Eurasian common shrew (*Sorex araneus*), alpine shrew (*S. alpinus*), Eurasian water shrew (*Neomys fodiens*), and common mole (*Talpa europea*) (*3*). Recently, genetically distinct hantavirus sequences have been found by reverse transcription–PCR in the Therese shrew (*Crocidura theresae*) in Guinea (*4*) and the northern short-tailed shrew (*Blarina brevicauda*) in the United States (*5*). In addition, a phylogenetically distinct hantavirus has been isolated from lung tissues of the Ussuri shrew (*C. lasiura*), captured along the Imjin River near the demilitarized zone in South Korea (J.-W. Song and R. Yanagihara, unpub. data).

## The Study

To further investigate the existence and phylogeny of nonrodentborne hantaviruses, we analyzed lung and other visceral tissues, collected in RNAlater Stabilization Reagent (QIAGEN, Valencia, CA, USA), from 24 soricomorphs, including 9 white-toothed shrews (*Crocidura* spp.), 3 Chinese mole shrews (*Anourosorex squamipes*), and 12 long-nosed moles (*Euroscaptor longirostris*), captured in northern, central, and southern Vietnam during November and December 2006. RNA, extracted from 20–50 mg of each tissue by using the RNA-Bee isolation kit (TEL-TEST, Inc., Friendswood, TX, USA), was reverse transcribed by using Moloney murine leukemia virus reverse transcriptase (Promega, Madison, WI, USA) and the primer 5′-TAGTAGTAGACTCC-3′. Oligonucleotide primers for subsequent nested PCR were designed from consensus regions of TPMV and other hantaviruses (Table 1).

**Table 1:** Oligonucleotide primers for amplification of Cao Bang virus

Segment	Primer	Sequence (5′→3′)	Polarity
Small	OSM55	TAG TAG TAG ACT CC	+
	OSM47	GGC CAG ACA GCA GAT TGG	+
	CBS1063F	ATK GCA TCH AAR ACA GTN GGN A	+
	CBS1016F	GGA GRA CWC AAT CAA TGG GT	+
	CBS1195F	GCN TGG GGN AAR GAG GCW GT	+
	CBS593R	GAC TGG GCA TTN GGC ATN GA	–
	CBS506R	ATH CTT GTC CCY TTR TTA TC	–
	S6	ACG TCI GGA TCC ATI TCA TC	–
	CBS-3′endR	TAG TAG TAK RCT CCY TRA A′	–
Medium	OSM55	TAG TAG TAG ACT CC	+
	G1–1	TAG TAG TAG ACT CCG CAA	+
	OSV697	GGA CCA GGT GCA DCT TGT GAA GC	+
	G2F1	TGG GCT GCA AGT GC	+
	CBM2762F	GGN AAY AHN GTC TCA GGN TAT	+
	CBM2804F	GAT TCH TTY CAA TCA TTY AA	+
	CBM2858F	GAR TGG GNA GAT CCW GAT	+
	CBM479R	AND TTG CAN GCA TGA ATA GG	–
	CBM505R	CCA ATS CAA NMA KAC AGC TT	–
	CBM1272R	TTH TGY TTW GAN ACA AGG CA	–
	CBM1322R	CHA CTC TYT GRC AMA CAA A	–
	T-M1442R	CCA TGN AAN CCT GGA ACA CA	–
	T-M1485R	CCA GCC AAA RCA RAA TGT	–
	CBM2256R	CAN GCM CCA TAR CAA TGA AA	–
	T-M2957R	GAA CCC CAD GCC CCN TCW AT	–
	G2T	TAG TAG TAK ACW CCG CA	–
Large	OSM55	TAG TAG TAG ACT CC	+
	PHL-173F	GAT WAA GCA TGA YTG GTC TGA	+
	PHL-2111F	CAG TCW ACA RTT GGT GCA AGT GG	+
	PHL-2935F	YTM ATG TAT GTT AGT GCA GAT GC	+
	TPMV-L195R	TTR TCA GAC CAD TCA TG	–
	TPMV-L345R	TRT AAT TRT CAG GTG T	–
	PHL-3445R	GRT TAA ACA TAC TCT TCC ACA TCT C	–
	PHL-3388R	AAA CCA TTC AGT TCC ATC ATC	–

Gene-amplification reactions were performed in 50-μL reaction mixtures, containing 200 μmol deoxyribonucleoside triphosphate, 0.5 U of super-therm polymerase (PureTech Co., Ltd, Seoul, South Korea), 1 μg of cDNA, and 10 pmol of each primer. Initial denaturation, at 94°C for 5 min, was followed by touchdown cycling with denaturation at 94°C for 40 s, annealing from 50°C to 37°C for 40 s, elongation at 68°C for 1 min 20 s, then 25 cycles of denaturation at 94°C for 40 s, annealing at 40°C for 40 s, and elongation at 68°C for 1 min 20 s in a Mastercycler ep gradient S (Eppendorf AG, Hamburg, Germany). PCR products were purified by the Wizard PCR Preps DNA Purification System (Promega). DNA sequencing of at least 3 clones of each amplicon was performed in both directions with the dye primer cycle sequencing ready reaction kit (Applied Biosystems, Foster City, CA, USA) on an automated sequencer (Model 377, Perkin Elmer Co., Waltham, MA, USA) (*6*).

Hantavirus sequences were not detected in tissues of the white-toothed shrews and long-nosed moles. By contrast, the full-length 3,637-nt (1,139-aa) medium (M) segment was amplified from lung tissues of 3 Chinese mole shrews, captured in Thanh Cong commune, Nguyen Binh District, Cao Bang Province, along the southern border of the People’s Republic of China. Designated Cao Bang virus (CBNV), the newly identified hantavirus exhibited low nucleotide and amino acid sequence similarity to representative hantaviruses harbored by Murinae, Arvicolinae, Neotominae, and Sigmodontinae rodents, ranging from 62.6% (nt) and 61.2% (aa) for Hantaan virus (HTNV) 76–118; 62.3% (nt) and 61.7% (aa) for Dobrava virus (DOBV) Greece to 58.1% (nt) and 52.0% (aa) for Puumala virus Sotkamo; and 58.8% (nt) and 54.7% (aa) for Sin Nombre virus NMH10 (Table 2).

**Table 2:** Pairwise nucleotide and amino acid sequence analysis of the full-length M segments of Cao Bang virus and other hantaviruses*

Hantavirus	CBNV	HTNV	SEOV	SOOV	DOBV	PUUV	PHV	TULV	SNV	ANDV
CBNV TC-3	–	62.6	62.4	62.5	62.3	58.1	57.3	58.3	58.8	58.0
HTNV 76–118	61.2	–	71.3	80.6	70.7	58.0	57.9	58.8	57.4	57.6
SEOV 80–39	60.9	77.1	–	71.1	70.4	58.6	57.5	58.8	56.7	57.2
SOOV SC-1	60.8	91.2	76.6	–	70.4	57.8	58.1	59.4	58.1	57.4
DOBV Greece	61.7	77.3	77.2	60.8	–	58.3	57.1	58.0	58.3	57.6
PUUV Sotkamo	52.0	53.8	53.8	54.1	53.4	–	70.1	71.4	65.5	64.8
PHV PH-1	52.4	54.3	53.6	54.2	53.5	75.6	–	73.0	65.5	63.6
TULV M5302v	52.9	55.7	54.7	54.8	55.0	78.9	80.4	–	66.4	64.8
SNV NMH10	54.7	54.7	53.1	55.2	54.1	66.9	67.6	69.2	–	70.6
ANDV Chile	54.2	54.7	54.1	55.3	54.4	66.8	66.7	67.8	77.9	–

*Pairwise distances are presented as a triangular matrix, with nucleotide on the top half and amino acid similarities on the bottom half. GenBank accession nos.: Cao Bang virus (CBNV TC-3), EF543526; Hantaan virus (HTNV 76–118), NC_005219; Seoul virus (SEOV 80–39), NC_005237; Soochong virus (SOOV SC-1), AY675353; Dobrava virus (DOBV Greece), NC_005234; Puumala virus (PUUV Sotkamo), NC_005223; Prospect Hill virus (PHV PH-1), X55129; Tula virus (TULV Moravia 5302v), NC_005228; Sin Nombre virus (SNV NMH10), NC_005215; and Andes virus (ANDV Chile-9717869), NC_003467.

Pairwise alignment and comparison of a 1,185-nt coding region of the small (S) segment showed similar degrees of sequence identity between CBNV and rodentborne hantaviruses, ranging from 61.4% for HTNV 76–118 to 58.0% for TULV (Tula virus) M5302v. Much higher sequence similarity was found in a 412-nt coding region of the large (L) segment, ranging from 72.6% for HTNV 76–118 and 75.2% for DOBV Greece, to 67.2%–70.1% and 67.7%–71.6% for hantaviruses harbored by Arvicolinae, Neotominae, and Sigmodontinae rodents, respectively. CBNV sequences were similarly divergent from Tanganya virus (TGNV) Tan826: for S segment, 63.5% (nt) and 65.0% (aa) similarity; for L segment, 71.3% (nt) and 77.0% (aa) similarity.

Phylogenetic trees based on sequences of the full-length M segment and partial S and L segments, generated by the maximum likelihood and neighbor-joining methods using the GTR+I+G model of evolution, showed similar topologies supported by bootstrap analysis, in which CBNV was relatively distinct from rodentborne and other shrewborne hantaviruses (Figure). A strong association with TGNV was observed on the basis of the S segment (1,185 bases), however. Further sequence information will clarify the relationship between CBNV and other soricidborne hantaviruses and whether these form a monophyletic group in parallel with the evolution of Soricinae and Crocidurinae shrews. If one judges by the distant evolutionary relationship between shrews and rodents, future sequences of other soricidborne hantaviruses will provide considerable insights into their evolutionary origins.

**Figure:**
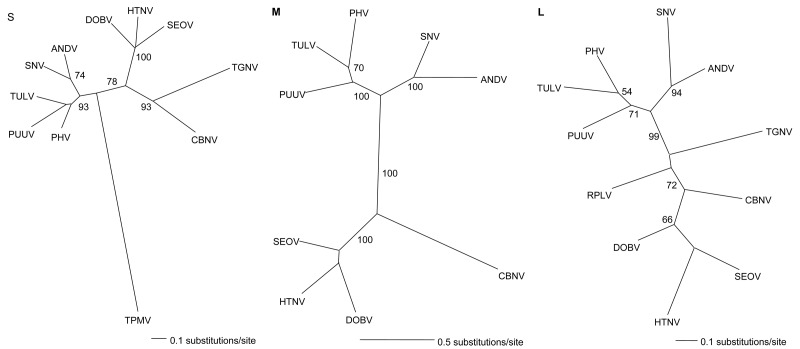
Phylogenetic trees based on the 1,185-nt partial small (S), 3,637-nt full-length medium (M), and 412-nt partial large (L) segments of Cao Bang virus (CBNV). The depicted S tree was generated by the neighbor-joining (NJ) method, by using the GTR+I+G model of evolution as estimated from the data. The M and L trees were generated by the maximum likelihood (ML) method, using the same model of evolution. The phylogenetic position of CBNV is shown in relationship to representative Murinae rodentborne hantaviruses, including Hantaan virus (HTNV 76–118, GenBank accession nos. NC_005218, NC_005219, NC_005222), Dobrava virus (DOBV Greece, GenBank accession nos. NC_005233, NC_005234, NC_005235), and Seoul virus (SEOV 80–39, GenBank accession nos. NC_005236, NC_005237, NC_005238); Arvicolinae rodentborne hantaviruses, including Tula virus (TULV M5302v, GenBank accession nos. NC_005227, NC_005228, NC_005226), Prospect Hill virus (PHV PH-1, GenBank accession nos. Z49098, X55129, EF646763) and Puumala virus (PUUV Sotkamo, GenBank accession nos. NC_005224, NC_005223, NC_005225); and Sigmodontinae and Neotominae rodentborne hantaviruses, including Andes virus (ANDV Chile 9717869, GenBank accession nos. NC_003466, NC_003467, NC_003468) and Sin Nombre virus (SNV NMH10, GenBank accession nos. NC_00521, NC_005215, NC_005217). Also included are Thottapalayam virus (TPMV VRC-66412, GenBank accession no. AY526097), Tanganya virus (TGNV Tan826, GenBank accession nos. EF050454, EF050455), and Camp Ripley virus (RPLV MSB89863, GenBank accession no. EF540771). NJ, ML, and maximum parsimony phylogenetic methods yielded similar topologies with only minor cosmetic differences. Host identification was confirmed by diagnostic mitochondrial DNA sequences (GenBank accession no. EF543528). The numbers at each node are bootstrap probabilities (expressed as percentages), as determined for 1,000 (NJ) and 100 (ML) iterations by PAUP* version 4.0 (Sinauer Associates, Inc. Publishers, Sunderland, MA, USA) (http://paup.csit.fsu.edu). GenBank accession nos. for CBNV: S (EF543524); M (EF543526); and L (EF543525).

## Conclusions

Designing suitable primers for the amplification of CBNV presented unanticipated challenges. Ironically, the recently acquired full genome of TPMV (J.-W. Song and R. Yanagihara, unpub. data) was not particularly helpful, since CBNV was genetically more divergent from TPMV than from well-characterized rodentborne hantaviruses (78% bootstrap support, S segment). Also, because the tissues were collected in RNAlater, virus isolation attempts could not be performed. As such, progress in obtaining the full-length sequence of CBNV has been slow.

A forest-dwelling soricine typically residing at elevations of 1,500–3,000 m, the Chinese mole shrew (family Soricidae, subfamily Soricinae) has a vast geographic range, extending from western and central People’s Republic of China, northern Myanmar, northern Thailand, Assam, Bhutan, northern Vietnam, Taiwan, and possibly Lao People’s Democratic Republic. That a hantavirus has been identified in the Chinese mole shrew was not completely unexpected, in view of the isolation of a HTNV-like virus from this species in Sichuan Province in 1986 (*7*). However, those authors may have prematurely concluded that their hantavirus isolate was closely related to HTNV, since no genetic analysis was performed.

Viewed within the context of newly identified, genetically distinct hantaviruses in the northern short-tailed shrew (*B. brevicauda*), Eliot’s short-tailed shrew (*B. hylophaga*), masked shrew (*Sorex cinereus*), and montane shrew (*S. monticolus*) in the United States (S. Arai and R. Yanagihara, unpub. data)—as well as in the Eurasian common shrew in Switzerland, Hungary, and Finland (J.-W. Song, S. Arai, and R. Yanagihara, unpub. data), the Ussuri shrew in South Korea (J.-W. Song and R. Yanagihara, unpub. data), the Asian house shrew in India (*1*,*2*), and the Therese shrew in Guinea (*4*)—the detection of a newfound hantavirus in the Chinese mole shrew would predict that hantaviruses harbored by shrews are as geographically widespread as those harbored by rodents. Preliminary studies indicate CBNV-like sequences in the liver tissue of Chinese mole shrews captured in Taiwan (S. Arai and R. Yanagihara, unpub. data). Also, investigations on the genetic diversity of CBNV and other newly identified members of the *Hantavirus* genus will provide additional insights into the phylogeography and co-evolution of hantaviruses and their soricid reservoir hosts. One or more of these newfound shrewborne viruses may yield valuable clues about the molecular determinants of hantavirus pathogenesis.
